# Emergence of Structural Patterns in Neutral Trophic Networks

**DOI:** 10.1371/journal.pone.0038295

**Published:** 2012-08-10

**Authors:** Elsa Canard, Nicolas Mouquet, Lucile Marescot, Kevin J. Gaston, Dominique Gravel, David Mouillot

**Affiliations:** 1 Institut des Sciences de l'Evolution, Université Montpellier 2, Montpellier, France; 2 Ecologie des systèmes marins côtiers, Université Montpellier 2, Montpellier, France; 3 Centre d'Ecologie Fonctionnelle et Evolutive, Centre National de la Recherche Scientifique, Montpellier, France; 4 Environment and Sustainability Institute, University of Exeter, Penryn, Cornwall, United Kingdom; 5 Département de biologie chimie et géographie, Université du Québec à Rimouski, Rimouski, Québec, Canada; King Abdullah University of Science and Technology, Saudi Arabia

## Abstract

Interaction networks are central elements of ecological systems and have very complex structures. Historically, much effort has focused on niche-mediated processes to explain these structures, while an emerging consensus posits that both niche and neutral mechanisms simultaneously shape many features of ecological communities. However, the study of interaction networks still lacks a comprehensive neutral theory. Here we present a neutral model of predator-prey interactions and analyze the structural characteristics of the simulated networks. We find that connectance values (complexity) and complexity-diversity relationships of neutral networks are close to those observed in empirical bipartite networks. High nestedness and low modularity values observed in neutral networks fall in the range of those from empirical antagonist bipartite networks. Our results suggest that, as an alternative to niche-mediated processes that induce incompatibility between species (“niche forbidden links”), neutral processes create “neutral forbidden links” due to uneven species abundance distributions and the low probability of interaction between rare species. Neutral trophic networks must be seen as the missing endpoint of a continuum from niche to purely stochastic approaches of community organization.

## Introduction

Ecological networks such as food-webs, pollination or host-parasitoid systems are among the most complex ecological systems. They exhibit structural patterns with key characteristics that give important information on their underlying ecological mechanisms (e.g. [1]–[3]). Early studies of trophic networks have used a variety of indices to characterize these patterns, including the number of interactions or links (*L*), link density (i.e. number of links per species, *D = L/S*) and connectance (proportion of links realised compared to all potential links, *C = L/S^2^*). Many scale-invariant patterns have been found and debated (e.g. the relative proportions of primary producers, secondary consumers and tertiary consumers; omnivory; intervality; cycling/looping - see for example [4]–[7]).

Among these patterns the density of links in networks has been of central interest as it is associated with the complexity-stability relationship [8], [9]. Two major complexity-diversity scaling laws have been proposed: in the first case, consumers are constrained to a constant number of interactions (“link species scaling law” [7]), while in the second they are constrained to feed on a constant proportion of the species pool (“constant connectance” [10]). These scaling laws have been extensively tested, and the current consensus is that observed patterns take an intermediate form [11], [12]. Beyond complexity-diversity relationships, two other patterns, related to the structure of links among species, have particularly been investigated during the past decade. Nestedness measures to what extent interactions by specialists are nested within those realized by generalists [13], and modularity measures the compartmentalization of the network into relatively independent community modules (e.g. [8], [14]). A nested interaction structure is hypothesized, at least, to buffer communities against extinctions or temporal fluctuations in the abundance of specialist species [15], [16] and to promote species coexistence [17]. Modularity is hypothesized to increase the stability of interaction networks since disturbances are less likely to spread across different modules that are weakly connected ([8]; see however [18]).

Many attempts have been made to elucidate the processes responsible for these observed patterns (e.g. [1], [6]). Most of the models used, either phenomenological or population based (*sensu* [1]), have in common that they assume ecological niche differentiation. The niche is often represented by body size [7], but also by other traits such as foraging behaviour [19] or approximated by phylogeny [20]. The importance of the niche concept has however been challenged by the neutral theory of biodiversity [21] and this perspective has yet to be integrated into the study of ecological networks. The neutral theory posits that speciation, dispersal, and demographic stochasticity are the main processes responsible for community structure [21], [22]. Using this parsimonious approach, common ecological patterns such as the shape of species–abundance distributions, species–area relationships, and distance decay in community similarity have all been found without invoking niche differences among species (e.g. [21]). Beside their intrinsic interest (e.g. [23]–[26]), neutral models provide an adequate null hypothesis to the niche theory [27], [28].

A neutral network assembly theory should help in disentangling the complexity of ecological interactions [29], [30], either by providing a realistic explanatory framework or at least an adequate null model. A niche-based network perspective considers species interactions to be constrained by specific functional traits (e.g. [31]), while a neutral one would only consider random interactions among individuals. Some previous studies have integrated randomness into mutualistic networks either as a null hypothesis [32] or as a neutral based model [29]. Solé *et al.* [9] also simulated population dynamics from random networks. Those pioneering studies found that a decreasing connectance with species richness, nested and modular structures could emerge from neutral assembly and interactions. However, these approaches have essentially been phenomenological and have thus not explicitly explored the simultaneous effects of neutral dynamics and neutral interactions on network structure.

In this study we simulate bipartite predator-prey networks (two trophic levels) with explicit neutral dynamics within each trophic level (neutral competitive interactions) and between levels (neutral predatory interactions). We investigate the structure of the emerging neutral networks by calculating species diversity, species relative abundance, density of links, connectance, nestedness, and modularity at equilibrium, and we compare our results with empirical values from the literature. We find that patterns of connectance and the complexity-diversity relationship in simulated neutral networks mimic those observed in empirical interaction networks. We also find that neutral networks may be nested and modular like those found in bipartite empirical networks. We then show how, within this neutral context, realistic network structure emerges simply from combining the patterns of relative species abundances found at each trophic level. Neutral dynamics create “neutral forbidden links” responsible for realistic ecological network complexity and organisation.

## Materials and Methods

### Model description

We simulated the dynamics of neutral bipartite predator-prey networks, in which individuals belonging to the higher trophic level (i.e. predators) consume any individuals of the lower trophic level (i.e. prey). Intra-guild predation is not allowed. We considered a single local community connected to a large metacommunity. Neutrality occurs at two levels: (i) at the community level we assumed ecological equivalence between individuals and species [21], and (ii) at the network level we assumed equivalence in the potential diet of individuals and species, i.e. any predator individual can feed on any prey individual with the same probability (a neutral interaction).

Three parameters determine the dynamics within each trophic level: reproduction rate *f*, immigration rate *m*, and carrying capacity as the number of individuals *K*. Initially, the prey and predator communities are composed of *K_prey_* and *K_pred_* individuals of a single species, respectively. Then the dynamical procedure iterates the following four actions at each time step: (i - interactions) each predator individual consumes a prey individual at random and the chosen prey individual dies (which approximates the case of a host-parasitoid network); (ii - birth) each individual gives rise to a single offspring with probability *f*; (iii - immigration) for each birth event, there is a probability *m* that the offspring is replaced by an immigrant, i.e. from a species not found in the local community but from an infinite regional species pool with a uniform abundance distribution as in Bell [22]; and (iv – density regulation) if the community exceeds its carrying capacity, individuals in excess are removed at random [21], [22].

### Parameters and simulations

We simulated communities with carrying capacities of 5000 prey and 1000 predator individuals, corresponding to a scenario in which predators are larger bodied and thus have lower abundances than their prey [33]. We also simulated communities with alternative carrying capacities of prey and predators to assess the robustness of our results to different parameter values (see Text S4). Birth rates were fixed at *f_pred_* = 0.2 for predators and *f_prey_* = 0.4 for prey. The birth rate was twice as high for prey as for predators, reflecting the accompanying differences in body size [34].

We simulated different immigration rates to vary species richness and abundance distributions (immigration rates were set equal for prey and predators and ranged between 1.10^−5^ and 1.10^−2^). This range ensured a sufficient equilibrium species richness without too many species having unrealistically short life spans [35]. We first simulated the model for 100 000 time steps and determined the number of steps that were sufficient to reach an asymptote in community structure. We then carried out 30 replicated runs of 10 000 time steps for each immigration rate.

### Network properties

During each simulation, the species richness of prey (*S_prey_*) and predator communities (*S_pred_*) and abundances were recorded. Evenness (*E_prey_* and *E_pred_*) of each community was estimated using Pielou's index [36].

The matrix of interactions *M(t)* (also called the matrix of links) is a *S_prey_*×*S_pred_* sized matrix summarizing the realized links (presence/absence) between each pair of predator and prey species at a given time step. *M(t)_i_*_,*j*_ = 1 when at least one individual of predator species *j* had consumed at least one individual of prey species *i,* and *M(t)_i_*_,*j*_ = 0 otherwise. This matrix was used to compute the following descriptors of network complexity: (i) the number of realized links in the network *L* (the sum of *M(t)*); (ii) the link density *D*, the ratio between the number of links and number of predator species (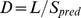); and (iii) connectance *C*. We used directed connectance, defined as the proportion of realized links among the total number of potential links [37], i.e. 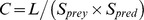
for bipartite acyclic networks.

We fitted the power law relationship between the number of realized links and species richness, 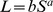. This relationship allows a direct comparison of the two alternative complexity-diversity relationships. An exponent of a = 1 yields the link species scaling law, where *L/S* is invariant to *S*. An exponent of a = 2 yields constant connectance, where *L/S* scales linearly with *S*. Note that the number of potential links is *S* ^2^ in multi-trophic networks, but *S_prey_*×*S_pred_* in bipartite acyclic networks. Consequently, we corrected the power law for bipartite networks by using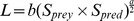. We combined all simulated networks (30 replicates for each immigration rate) to estimate the slope of the complexity-diversity relationship. We used a type II Standardized Major Axis (SMA) regression [38] because there was error variance in both the independent (*L*) and the dependent (*S_prey_*×*S_pred_*) variables. Second, we compared this relationship and that found within replicates for each immigration rate (8 slopes), using a Bartlett-corrected likelihood ratio statistic. All tests were conducted with R [39].

We estimated the nestedness of interactions within each bipartite network [13] using the function ‘nestedness’ of the ‘bipartite’ package for R [40]. This function calculates the nestedness temperature *T* of the interaction matrix by analogy to physical disorder. To emphasize nestedness instead of disorder, we used the nestedness *N* that is usually expressed as *N* = (100−*T*)/100, with values ranging from 0 to 1 (maximum nestedness). We estimated the modularity *Q* of interactions using an index developed for bipartite networks [41]:
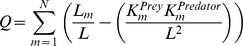
where *N* is the number of modules in the network, *L* the total number of links, *L_m_* the number of links between all the species within module *m*, and 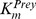 and 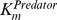 are respectively the sum of the number of links of all the prey and all the predators in module *m*. This index was maximized with a simulated annealing algorithm following the method developed by Guimera and Amaral (see Methods in [42], ).

Because nestedness and modularity measures are potentially affected by the number of species and the number of links within the network [44], , we also estimated relative nestedness and modularity [13], [29]. The relative metrics calculate how nested and modular is the matrix of interactions when compared with expectations from a null model, and they allow comparisons between networks of different size and connectance values [13]. The relative nestedness N* is
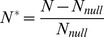
where *N* is the observed nestedness and *N_null_* is the mean nestedness value over 100 replicates of the null model. For each simulated neutral network, we calculated the relative nestedness using a null model that conserved the number of species and the degree distributions of each trophic level, as describe in Bascompte *et al.* [13]. We proceed the same way for the relative modularity Q*.

## Results

### Dynamic equilibrium of network complexity and structure

**Figure 1:**
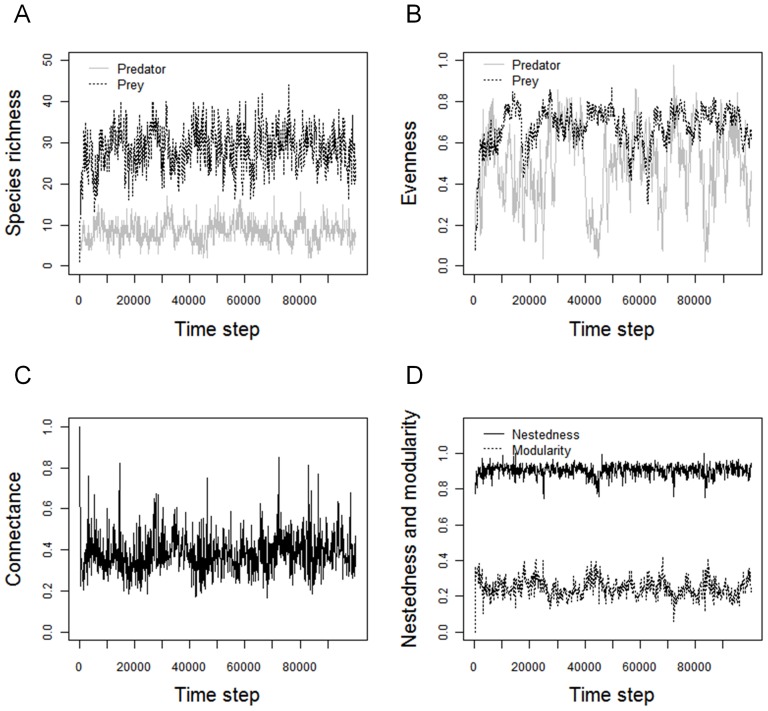
Example of a network assembly simulation through time for *m* = 10^−3^. Prey (dashed black line) and predator (grey line) community (A) species richness and (B) evenness are shown, and network (C) connectance, and (D) nestedness and modularity.

An example of the temporal dynamics of network structure is illustrated in Figure 1 for *m* = 10^−3^. Species richness and evenness rapidly increased to reach equilibrium values (Figure 1A–1B, *S_prey_* = 29.0±4.7, *S_pred_* = 8.3±2.6, *E_prey_* = 0.69±0.08, *E_pred_* = 0.49±0.18, means calculated between 10 000 and 100 000 time steps), but evenness showed strong fluctuations with maximum values of 0.87 for prey and 0.98 for predators. Predator evenness showed stronger temporal variation due to the sensitivity of this index to species number (which was lower for predators). Connectance also rapidly reached equilibrium (Figure 1C, *C* = 0.38±0.09, means calculated between 10 000 and 100 000 time steps). We found patterns of temporal fluctuation due to ecological drift, but there were no limit cyclcological drift, but there were no limit cycles (see Text S1). Nestedness and modularity also rapidly reached equilibrium, and these values showed smaller fluctuations in time than the other indices (Figure 1D, *N* = 0.90±0.03, *Q* = 0.25±0.05).

### Effect of immigration rate on network complexity

**Figure 2:**
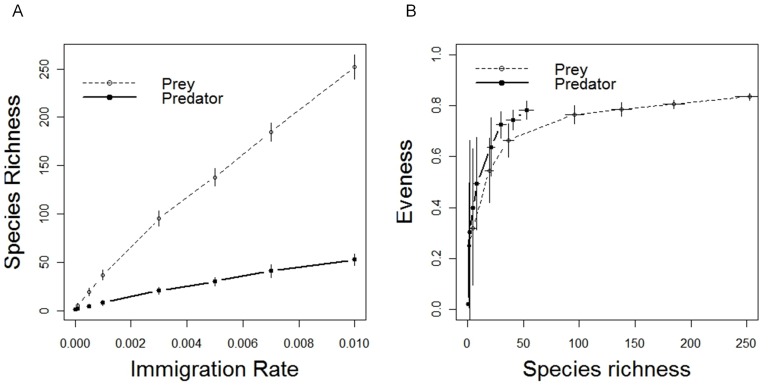
Diversity of prey (dashed black line) and predator (grey line) communities at equilibrium with increasing immigration rates. (A) Species richness as a function of immigration rate, and (B) evenness of predator and prey communities as a function of species richness at each trophic level. Total species richness is the total number of species in the network. Error bars represent standard deviation over the 30 replicates. Parameters are as described in the methods.

**Figure 3:**
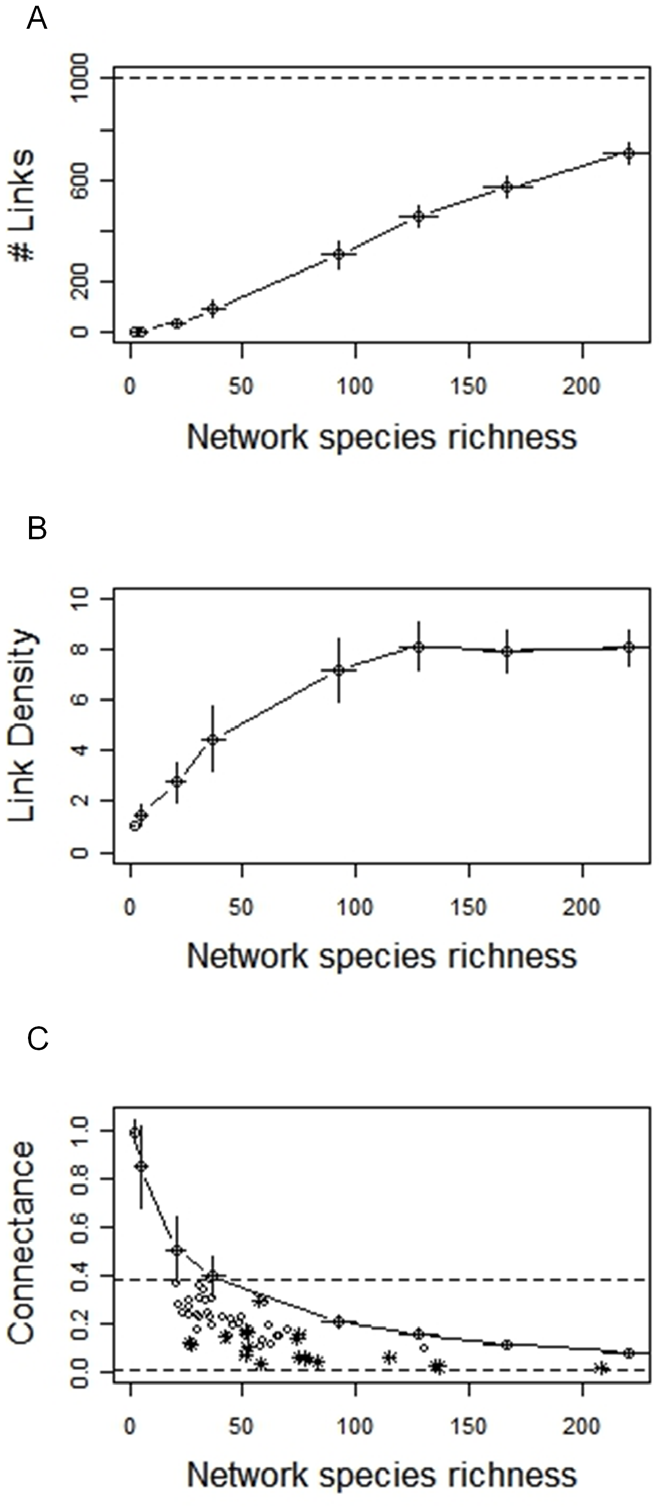
Link density structure as a function of total network species richness (*S_prey_*+*S_pred_*) at equilibrium. (A) Number of realized links (the upper horizontal dashed line is the maximum number of links that can be realized), (B) link density and (C) connectance. Error bars represent standard deviations over the 30 replicates of each immigration rate. Symbols correspond to empirical connectance values reported for host-parasite (circles) and predator-prey (stars) bipartite networks. Host-parasite network data were extracted from the meta-analysis of Fortuna and colleagues [44], and predator-prey data from Thébault & Fontaine study [43]. Dashed lines correspond to the extreme values of indices found for these networks. Connectance values of neutral networks fall within the range of values reported for empirical networks (host-parasite: 0.10–0.38 [44] and predator-prey: 0.01–0.30 [43]).

Network species richness increased with the immigration rate (Figure 2A). Predator and prey evenness saturated with species richness (Figure 2B). Network topological attributes showed contrasting tendencies: the number of realized links (Figure 3A) increased with network species richness while link density showed a hump-shaped relationship (Figure 3B). The number of realized links is expected to saturate for higher species richness values as the total number of links is constrained by the total number of predators (*K_pred_* = 1000, Figure 3A). Connectance values ranged between 0.04 and 1 (Figure 3C). Connectance values were close to 1 for very species poor communities (e.g. *S_pred_* = 1.03±0.18 at *m* = 10^−5^; *S_pred_* = 1.90±0.93 at *m* = 10^−4^) in which there were few potential links. Then, *C* decreased very abruptly as species richness increased.

### Complexity-diversity relationship

**Figure 4:**
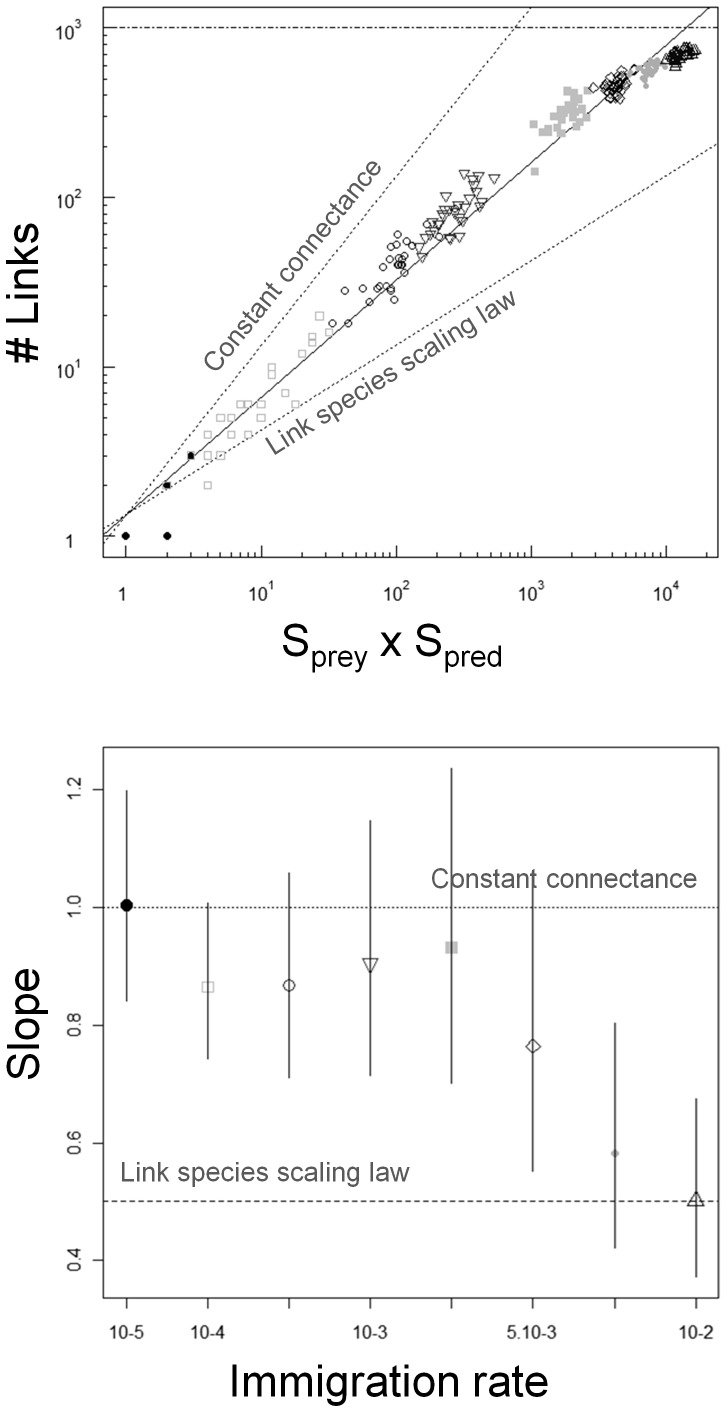
Relationship between complexity (number of links *L*) and diversity (*S_prey_*×*S_prey_*) at equilibrium for different immigration rates (*m* = 1.10^−5^ black filled circles; 1.10^−4^ empty squares; 5.10^−4^ empty circles; 1.10^−3^ inversed empty triangles; 3.10^−3^ filled grey squares; 5.10^−3^ empty diamonds; 7.10^−3^ filled grey circles; 1.10^−2^ empty triangles). (A) Log-log type II regression for all simulated networks (30 replicates×8 immigration rates). The slope of the regression is 0.697 (*R^2^* = 0.99, *p*<2.10^−16^). The upper horizontal dashed line corresponds to the maximum number of links that can be realized in the network. The upper dotted line corresponds to the constant connectance hypothesis (slope = 1) and the lower dotted line to the link species scaling law (slope = 0.5). (B) Slopes of the log-log type II regression calculated among the replicates of each immigration rate (error bars are 95% confidence intervals for each slope).

We found over all simulations a complexity-diversity relationship with a slope of 0.69 (log-log linear regression through all networks simulated, Figure 4A). However, the slope varied with the immigration rate (Figure 4B, *p* = 0.004, Bartlett-corrected likelihood ratio test). Values ranged between 1 and 0.5 and decreased as immigration rate increased (Figure 4B), indicating that the linear log-log relationship was lost over a large gradient of species richness.

### Effect of immigration rate on network structure

**Figure 5:**
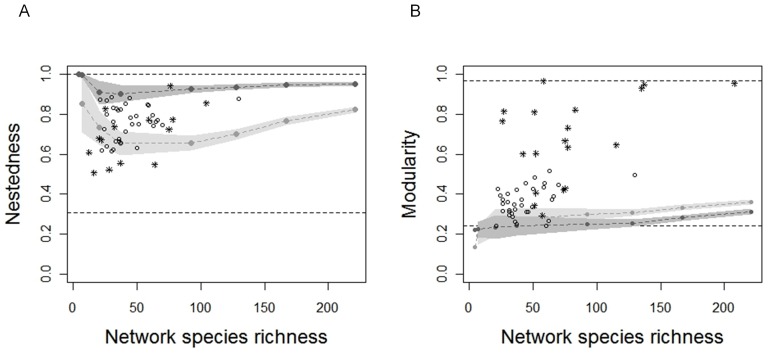
Network structure as a function of total network species richness (*S_prey_*+*S_pred_*) at equilibrium. (A) Nestedness and (B) modularity values for neutral (dark grey) and null (grey) networks. Grey zones represent intervals (mean ± standard deviation) over the 30 replicates for each immigration rate. Symbols correspond to empirical values reported for host-parasite (circles) and predator-prey (stars) bipartite networks. Host-parasite network data were extracted from the meta-analysis of Fortuna and colleagues [44]. Nestedness values of predator-prey networks were extracted from Bascompte *et al.* [13]. Modularity values of predator-prey networks were extracted from Thébault & Fontaine study [43]. Dashed lines correspond to the extreme values of indices found for those empirical networks. Nestedness and modularity values of neutral networks fall into the range of values reported for empirical networks (Nestedness: 0.507–0.942, with host-parasite: 0.613–0.886 and predator-prey: 0.507–0.942; Modularity: 0.241–0.965, with host-parasite: 0.241–0.518 and predator-prey: 0.291–0.965).

Nestedness values remained very high (ranging between 0.9 and 1, dark grey points, Figure 5A), independently of network species richness. Modularity was between 0.2 and 0.3, increasing lightly with species richness (dark grey points, Figure 5B). We found that neutral networks are more nested that expected under the null model (grey points, Figure 5A). However, modularity values of neutral networks did not differ from the null expectations for small size networks whereas they were lower than expected for larger networks (grey points, Figure 5B).

## Discussion

### Neutral networks structure

We found connectance values for neutral networks that ranged between 0.05 and 0.5 when total species richness was greater than 20 species (Figure 3C). These relatively low values for a neutral model illustrate how only a small fraction of potential interactions may be realized even in a network in which virtually all links are possible at any one moment. This range of connectance is consistent with values reported for real networks, particularly bipartite networks where these values vary between 0.01 and 0.38 [44]. Low connectance values are usually attributed to a restriction of interactions, also called “forbidden links” [5], because of niche incompatibilities that emerge from various constraints such as body size [11], morphology, phenology [46] and phylogeny [14], [47]. Here we show that low connectance can also be found in bipartite networks assembled without any niche constraint.

We also found that connectance decreased with species diversity, as commonly observed in empirical studies, including those done on the best resolved networks [11], [48], and in the stochastic models of Solé *et al.* [9]. We found a log-log complexity-diversity relationship with a slope of 0.69 (Figure 4A), equivalent to a slope of 1.38 in multi-trophic networks (see Methods). This value falls between the link scaling law (slope = 1) and that predicted by the constant connectance hypothesis (slope = 2), which is the actual consensus on complexity-diversity relationships for both niche assembly models [12], [48] and empirical data (for example 1.4 for the 113 webs catalog [7]; 1.4 for 50 pelagic lake food webs [49]; 1.73 for 12 highly resolved food webs [10]; 1.5 for 19 diverse food webs from a variety of habitat [6]).

We found both nested and modular patterns in neutral predator-prey networks (Figure 5). It has been suggested that nested patterns in food-webs are caused by species traits [50], for instance body size (interspecific hierarchy hypothesis as in the cascade model [7]) or specific resource qualities (the optimal diet choice theory [51]). As an alternative we suggest that neutral interactions may also produce both nested and modular patterns without any niche differentiation between species. We therefore conclude that the occurrence of nested and modular patterns cannot discriminate between neutral and non-neutral network organization. That said, we found that neutral networks were significantly more nested than expected under the null model (Figure 5), and that nestedness values were located in the upper limit of empirical values for antagonist networks. This is in accordance with Krishna *et al.* [29] who found that nestedness of a mutualist neutral network assembled with a phenomenological model was high in comparison to empirical networks. In addition, we found that neutral networks are lightly less modular than expected under the null model, with modularity values located at the lower limit of empirical values for antagonist networks. This suggests that neutral models of interaction networks tend to overestimate realized nestedness and underestimate realized modularity making deviations from neutral predictions potentially useful as measures of niche importance in structuring species interaction networks.

### Abundance patterns and neutral structure in networks

In our neutral perspective, individuals interact at random so that a species is more likely to interact with others when abundant. Not surprisingly, we found that both prey and predators had non–uniform species abundance distributions (see Text S2). Consequently, the probability that a given predator and a given prey species interact is highly variable. Especially, rare species make very improbable potential links in the network, that we call “neutral forbidden links”. For example, the probability with which a predator and a prey species that have each a relative abundance of 1% will encounter each other and interact is 0.01×0.01 = 0.0001. In other words, there will be on average 1 link per 10 000 predator individuals. To illustrate the relationship between patterns of relative species abundance distributions and network structures we simply calculated the expected matrix of species interactions from the equilibrium distributions of relative abundances for predator and prey communities obtained in our simulations (i.e. phenomenological model as in [1]; see Text S3). We found a strong correlation between the structures of networks assembled with the dynamical and the phenomenological model (Text S3, C: *r* = 0.99; *Q*: *r* = 0.95; *N*: r = 0.29). This confirms that food web structures within neutral networks mainly result from combining the relative abundance distributions found within each level.

The presence of neutral forbidden links clearly explains low connectance values in neutral networks, because it decreases the number of links effectively realized. Neutral forbidden links also explain nested patterns. Indeed, links mostly occur between abundant predator and prey species and they become rarer when at least one species becomes rare. In that way, a rare species is likely to realize a subset of the links realized by more abundant species within each trophic level, producing a nested distribution of links in the network. Nested patterns of interactions maximise species ‘niche overlap’ (i.e. shared interactions) which does not favor the emergence of independent modules. However, some modules still occur in neutral networks because of stochastic effects.

The occurrence of neutral forbidden links also explains why the slope of the complexity-diversity relationship varies with immigration. At low immigration rates, turnover is low, most species are abundant and any species is likely to realize a high number of links. The slope of the complexity-diversity relationship is elevated because there are only few neutral forbidden links. At high immigration rates however, there is a large number of rare and short-lived species realizing few links because of high species turnover [21], [35] (see Text S2). The slope of the complexity-diversity relationship is then much shallower, and species tend to have a constant (and maximum) number of links (as proposed by the link species scaling law). These results show how immigration and neutral drift, by shaping the abundance distribution of species, can produce either a single large-scale or different small-scale complexity-diversity relationships. This corresponds to what is observed in empirical networks which alternatively support the link scaling law, the constant connectance hypothesis or fall in between [11], [12].

Species relative abundance has already been proposed as one of several factors affecting network structure [30], [32], [52], [53]. Here we have provided a conceptual development to this proposition. Along with the phenomenological model proposed by Krishna *et al.* [29] for mutualism, our results can be seen as an endpoint of a continuum from network structures that are completely determined by species relative abundances (neutral model) to those in which niches entirely drive species interactions (niche model). Our main result is that neutral forbidden links could shape network structure, just as niche-mediated forbidden links do. We should expect that, even if they are neutral, links between abundant species occur consistently through time, while links between transient and rare species are much more ephemeral. Those weak links would be undetectable in the field or would require very intensive sampling [37], and can appear as niche forbidden links. A recent study by Dorado *et al.* [54] illustrates this limitation in nature, showing that rare species only realize a few links at a time and thus appear as specialists.

### Robustness of neutral forbidden links

Our approach promotes the occurrence of neutral forbidden links because we assumed that each predator individual feeds on a single prey individual at a time and we assessed the network structure in a snapshot. The concept is however robust to these assumptions. Neutral forbidden links occur because of the uneven relative abundance distribution of species rather than their absolute abundance. The first assumption means that a single species cannot realize more links than its absolute abundance. This constrains the network to a maximum number of links and consequently connectance. This upper bound on network complexity may also occur *in natura,* as the number of potential links does not account for constraints posed by species densities. Typically, connectance is calculated as L/S^2^. This metric does not take into account that some species are not abundant enough to realize all potential links, even if each individual could potentially feed on multiple preys. We tested the robustness of our results to the finite number of individuals by performing simulations with different sets of *K_pred_* and *K_prey_* (see Text S4). Varying *K_pred_* or *K_pred_*/*K_prey_* shifts the saturation of link number to higher values of species richness (Figure A in Text S4) but does not affect the complexity-diversity relationship (Figure B in Text S4). The analysis of network structure at a single moment in time also restricts network complexity. One might hypothesize that the number of links would accumulate over time until all species eventually interact with each other [55]. However, we found that when connectance is compiled over several time steps, even if it increases slightly, it remains at low values (see Text S5). This result supports the idea that some links between abundant species occur consistently through time, while the neutral forbidden links remain sporadic and thus do not contribute much to network structure. We also note that available data in the literature are commonly derived from restricted temporal windows, and thus record fewer links than would be found with more prolonged sampling efforts [56].

### Conclusion

The literature on interaction networks has largely ignored the implications of the neutral theory, despite its more widely recognized importance in structuring horizontal diversity [26], [57]. We have shown that a neutral network assembly model can lead to realistic patterns of network organization such as the complexity-diversity relationship, nestedness and modularity. Beyond the neutral assumption, our results suggest that the shape of species abundance distributions creates neutral forbidden links between species, which has profound impacts on network complexity and deserves as much attention as niche-mediated constraints. Along with Krishna *et al.* [29], we advocate considering neutral interactions as a potential driver of network dynamics. We do not argue that networks are organized exclusively by neutral processes, but instead that, just as neutral and niche processes can simultaneously shape the structure of competitive communities [23], [27], these two forces are likely to act together to shape the structure of ecological networks. The challenge is thus no longer to assess whether niche or neutral processes operate in networks but to disentangle their relative contributions [57]. This stresses the need for temporal data to assess the dynamics of network structure and to measure, for instance, the turnover of links over time. We view our neutral network assembly model as a useful null model, as any deviation from its predictions can be used to quantify the extent to which niche assembly processes constrain network organization.

## Supporting Information

Text S1 **Temporal autocorrelation of assembled network characteristics.**
(PDF)

Text S2 **Abundance distributions of prey and predator species in assembled neutral networks.**
(PDF)

Text S3 **Neutral phenomenological assembly model.**
(PDF)

Text S4 **Variation of complexity-diversity relationship with carrying capacity.**
(PDF)

Text S5 **Sensitivity of the network structure to accumulating interactions in time.**
(PDF)
