# Supplementary material for: Emergence of Structural Patterns in Neutral Trophic Networks
Source: PLoS One. 2012 Aug 10;7(8):e38295. doi: 10.1371/journal.pone.0038295 (PMC3416803; doi:10.1371/journal.pone.0038295)
Supplement: Text S1 — Temporal autocorrelation of assembled network characteristics. (PDF) [file pone.0038295.s001.pdf]

Supporting information S1: Temporal autocorrelation of assembled network characteristics

For each simulation we tested for temporal autocorrelation of community (S_{prey}, S_{pred}, E_{prey}, E_{pred}) and network (L, and C) characteristics by correlating values separated by lags of 1 to 10000 time steps (with the 'acf' function of the package 'stats' developed in the R package [1]).

For an immigration rate of $m = 10^{-5}$, we found that temporal autocorrelation (*acf*) of all descriptors (S_{prey}, S_{pred}, E_{prey}, E_{pred}, L, C) ranged between -0.09 and 0.14 (*acf* was between -0.094 and 0.105 for S_{prey}; -0.062 to 0.140 for S_{pred}; -0.062 to 0.127 for E_{prey}; 0.050 to 0.042 for E_{pred}; -0.117 to 0.108 for L; -0.076 to 0.126 for C; Figure S1), except for small lags (from 1 to 100). This result was expected because our model followed a process whereby state at time t depends on the state at time t-1. Temporal fluctuation patterns due to the stochastic nature of our neutral dynamics showed no sign of limit cycles.

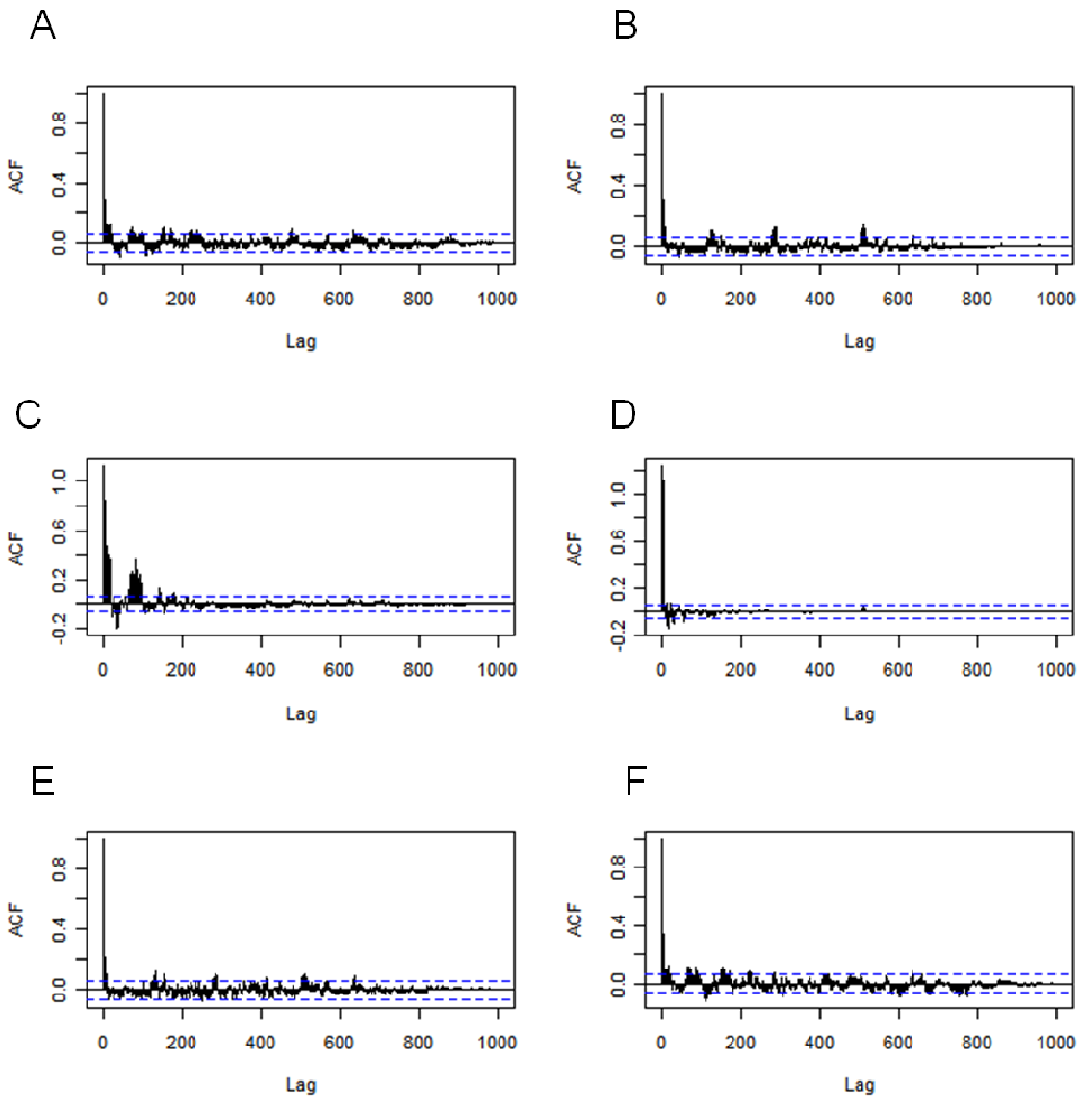

Figure S1. Estimates of temporal autocorrelation for one simulation example with m = 10^{-5}, for (A) prey species number S_{prey}, (B) predator species number S_{pred}, (C) prey abundance evenness E_{prey}, (D) predator abundance evenness E_{pred}, (E) number of realized links L, and (F) network connectance C.
