# Supplementary material for: Emergence of Structural Patterns in Neutral Trophic Networks
Source: PLoS One. 2012 Aug 10;7(8):e38295. doi: 10.1371/journal.pone.0038295 (PMC3416803; doi:10.1371/journal.pone.0038295)
Supplement: Text S2 — Abundance distributions of prey and predator species in assembled neutral networks. (PDF) [file pone.0038295.s002.pdf]

Supporting information S2: Abundance distributions of prey and predator species in assembled neutral networks

We illustrate the asymmetry between abundant and rare species within each of the two trophic levels. For this, we estimated the mean between the commonest species abundance and the rarest species abundance, and we reported the proportion of species in the community that contribute to the first part (abundant) and the last part (rare) of the abundance distribution. We used 100 replicates for three immigration rates (1.10^{-4}; 1.10^{-3}; 1.10^{-2}).

We found that, as immigration rate increases, a larger proportion of the community was composed by rare species (Figure S2).

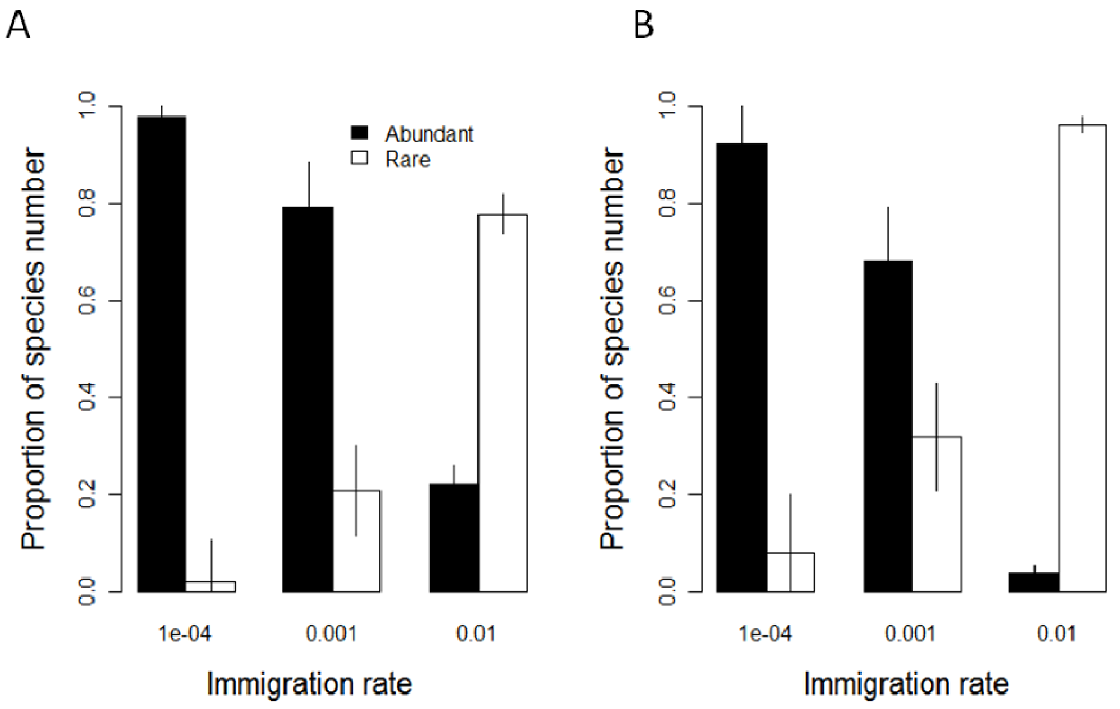

Figure S2. Asymmetry of the abundance distributions of species. Proportion of abundant (black) and rare (white) species in the (A) prey and (B) predator trophic level for three immigration rates (1.10^{-4}; 1.10^{-3}; 1.10^{-2}).
