# Supplementary material for: Emergence of Structural Patterns in Neutral Trophic Networks
Source: PLoS One. 2012 Aug 10;7(8):e38295. doi: 10.1371/journal.pone.0038295 (PMC3416803; doi:10.1371/journal.pone.0038295)
Supplement: Text S3 — Neutral phenomenological assembly model. (PDF) [file pone.0038295.s003.pdf]

Supporting information S3: Neutral phenomenological assembly model

To explore the effect of abundance patterns on network structure, we constructed a phenomenological assembly model [2]. This model simply simulates a matrix of species interactions (called M_{phe}) combining the equilibrium relative species abundance distribution of predator and prey communities given by the dynamical model. In our neutral perspective, species interactions result from the random encounter of predator and prey individuals [as in 3]. Thus the probability of interaction between a predator species j and a prey species i is simply the product of their relative abundance, i.e. $Pij = \frac{Ai}{Kprey} \times \frac{Aj}{Kpred}$, with A being the abundance of species, and K the carrying capacity of the community. As we assumed that each predator eats only one single prey at each time step, each predator species may realize as many links as their abundance. Consequently we considered that each predator species j realized A_j links following the probability of encounter P_{ij}. We then set $M_{phe}[i,j] = 1$ if species i and j realized at least one link, and $M_{phe}[i,j]=0$ otherwise.

We ran the neutral phenomenological assembly model to obtain 30 replications of M_{phe} with the parameters described in Methods. We conducted a correlation test between the connectance, nestedness and modularity values calculated from the phenomenological matrix (C_{phe}, N_{phe} and Q_{phe}) and those obtained from the dynamic matrix (C, N and Q).

We found that connectance and modularity values from the phenomenological matrix and those from the dynamic matrix were perfectly correlated (Connectance: r = 0.99, Figure Figure S3A; Modularity: r = 0.95, Figure S3C). This shows that connectance and modularity of networks assembled with the dynamic neutral model are the result of abundance patterns only. For nestedness we obtained a weaker correlation (r = 0.28, Figure S3B) suggesting the role of factors other than abundance distribution in the dynamic neutral model.

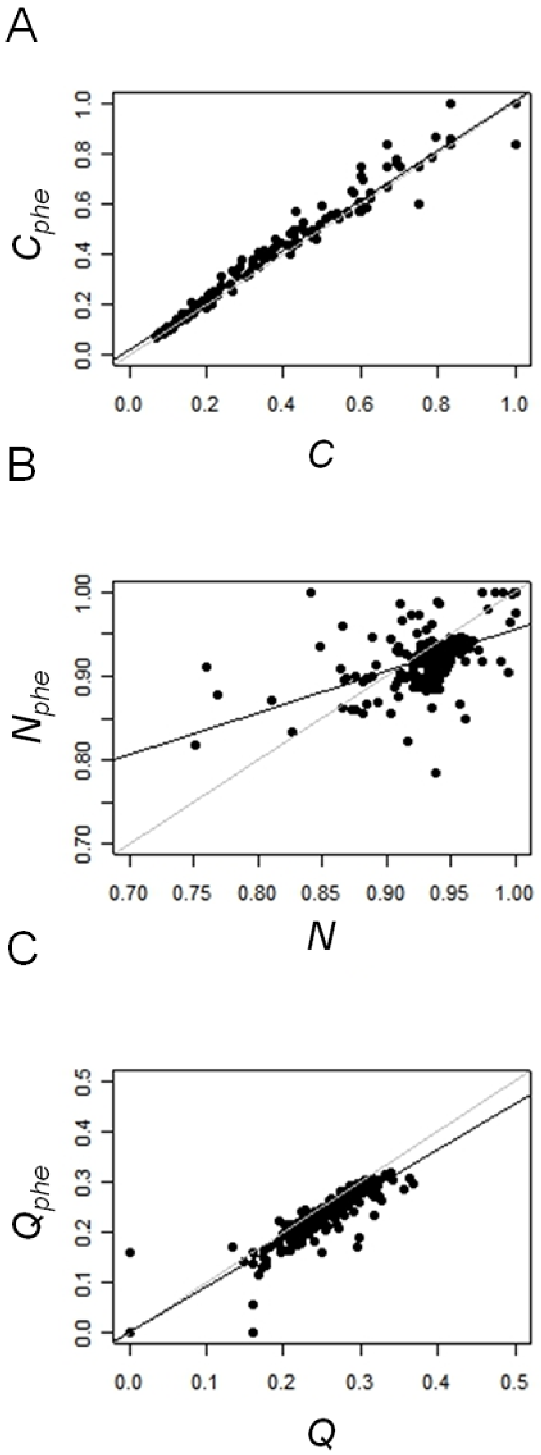

Figure S3. Correlation between (A) connectance, (B) nestedness, and (C) modularity values of networks assembled with the dynamic neutral model and with the phenomenological neutral model for diverse immigration rates (1.10^{-5}; 1.10^{-4}, 5.10^{-4}; 1.10^{-3}; 3.10^{-3}; 5.10^{-3}; 7.10^{-3}; 1.10^{-2}). Grey lines are 1:1 relation.
