# Supplementary material for: Emergence of Structural Patterns in Neutral Trophic Networks
Source: PLoS One. 2012 Aug 10;7(8):e38295. doi: 10.1371/journal.pone.0038295 (PMC3416803; doi:10.1371/journal.pone.0038295)
Supplement: Text S4 — Variation of complexity-diversity relationship with carrying capacity. (PDF) [file pone.0038295.s004.pdf]

Supporting information S4: Variation of complexity-diversity relationship with carrying capacity

We investigated the sensitivity of the complexity-diversity relationship to the carrying capacity. We first ran the dynamical assembly network model as described in the methods, for different predator carrying capacity K_{pred} values (500, 1000, 1500, 2000 and 2500 individuals). We maintained the predator/prey carrying capacity ratio equal to 1/5. We then ran the dynamical assembly network model for different predator/prey carrying capacity ratio values (1/4, 1/5, 1/6, and 1/7), and we maintained the predator carrying capacity K_{pred} equal to 1 000. For each set of simulations, we investigated the complexity-diversity relationship as explained in the methods.

We first found that the saturation of the relationship followed the maximum number of links for each carrying capacity value (Figure S4A). The slope of the regression was weakly influenced by the predator carrying capacity value (between 0.68 and 0.69, Figure S4A) or by the predator/prey carrying capacity ratio (between 0.67 and 0.70, Figure S4B), and falls between 0.5 and 1.

A

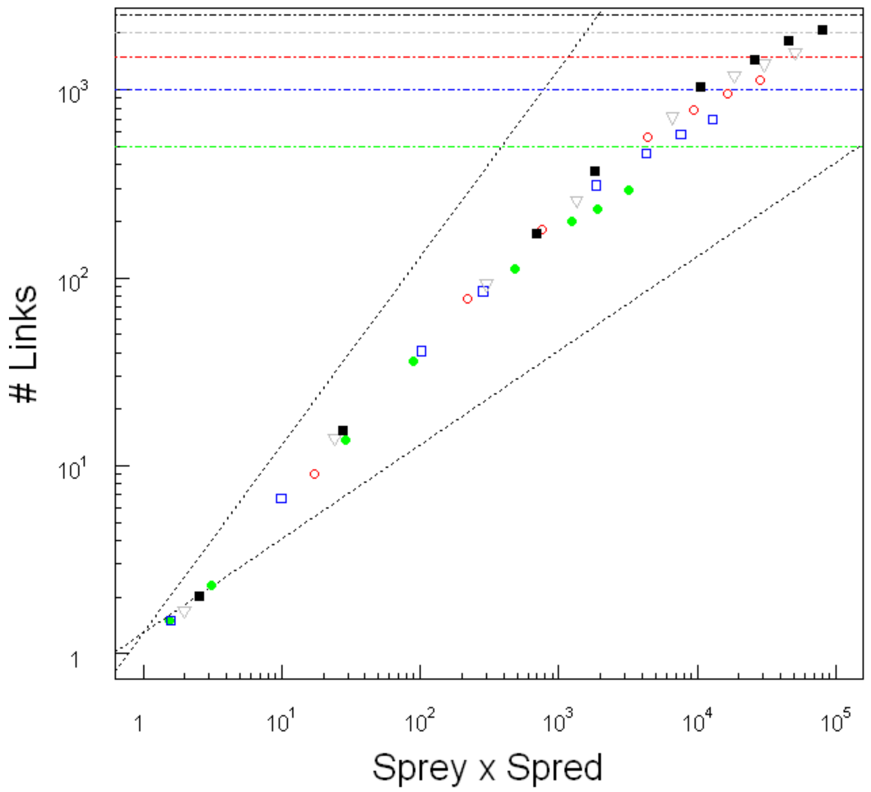

B

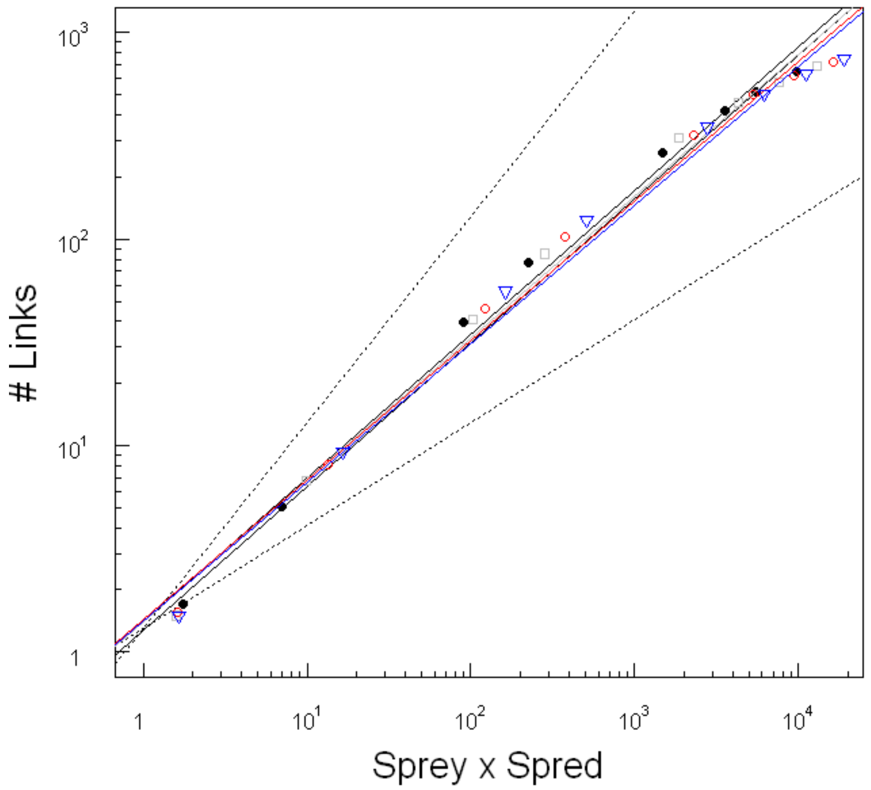

Figure S4. Variation of the complexity-diversity relationship with carrying capacity. (A) Complexity-diversity relationship for different predator carrying capacities K_{pred}. Different colors (green, grey, red, blue, black) correspond to different carrying capacities (500, 1000,

1500, 2000, and 2500). 30 replicates have been implemented for each immigration rate *m* value. (B) Complexity-diversity relationship for different predator/prey carrying capacity ratios. Different colors (black, grey, red, blue) correspond to different carrying capacity ratios (1/4, 1/5, 1/6, 1/7). 30 replicates have been implemented for each immigration rate *m* value.
