# Supplementary material for: Emergence of Structural Patterns in Neutral Trophic Networks
Source: PLoS One. 2012 Aug 10;7(8):e38295. doi: 10.1371/journal.pone.0038295 (PMC3416803; doi:10.1371/journal.pone.0038295)
Supplement: Text S5 — Sensitivity of the network structure to accumulating interactions in time. (PDF) [file pone.0038295.s005.pdf]

Supporting information S5: Sensitivity of the network structure to accumulating interactions in time

We assumed that predators feed on one single prey at each step, as would be the case in a host-parasitoid system. However, if we consider a time step as an event of predation, we could assume that a predator will be involved in several predation events by summing their prey items over several time steps. To evaluate the potential influence of eating several prey items on the network structure, we constructed such networks in which interactions are accumulated in periods of from 1 to 20 time steps after equilibrium. We compared network structure of those cumulated networks (connectance $C_{cum,}$ nestedness $N_{cum,}$ and modularity Q_{cum}) with snapshot network structures (C, N and Q) along the 20 time steps. 30 replicate simulations were made.

We found that connectance C_{cum} slightly increased with the cumulative period, and then stabilized. Connectance, although increasing, remains at low values. Two phenomena are influencing connectance values, with opposite effects when accumulating interactions in time: (i) Species increased their random interaction range with time because of sampling effects, thus time tends to increase connectance values. (ii) However, links involving abundant species are the most likely and would consistently occur through time, while the neutral forbidden links remain sporadic and thus do not contribute much to network structure. In addition, some species appeared only transiently in the network: new species entering the network because of immigration, and others which went extinct because of drift. These species interact poorly because of their rarity and their absence: they tend to decrease the connectance value.

The nestedness $N_{cum,}$ slightly increased with cumulative period, while modularity Q_{cum} tended to decrease especially for high immigration rates (10^{-2}). However, values stay in the range found for networks where only one prey is consumed at each time step. Those patterns

could be caused by an increase of niche overlap of species when they accumulate links through time that decrease the stochastic effect.

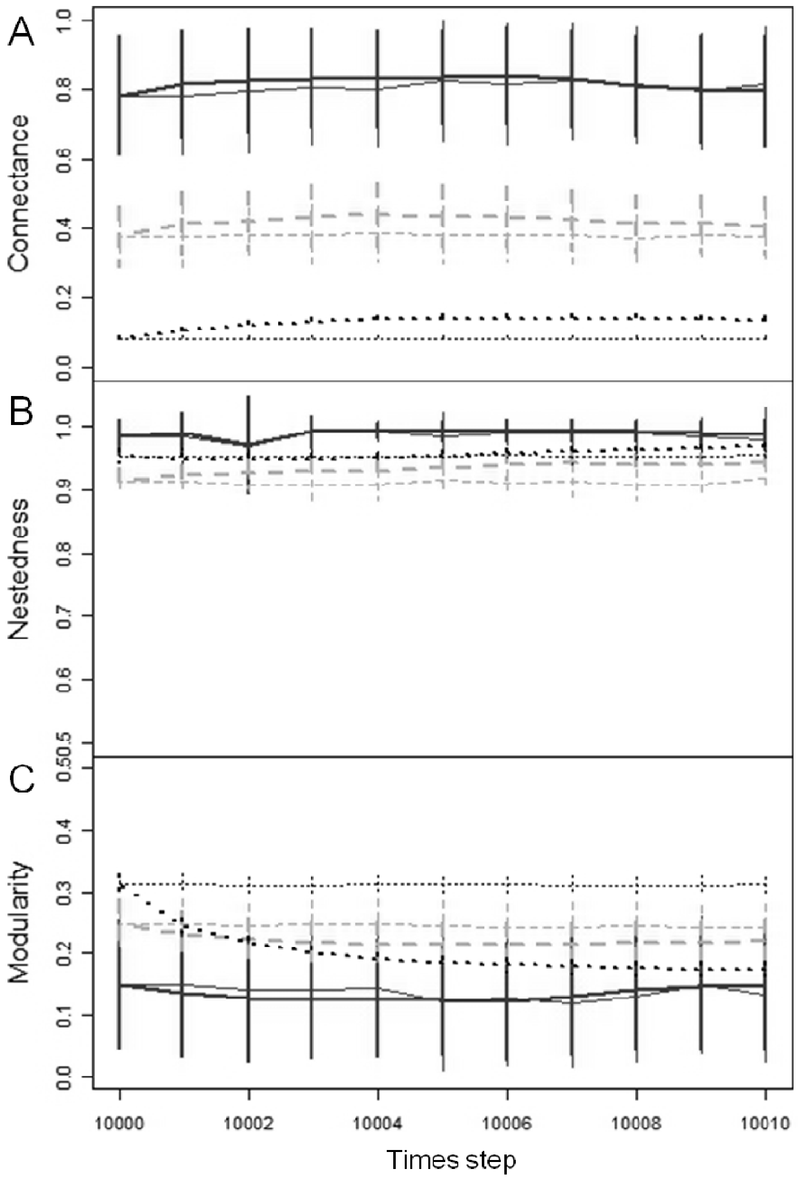

Figure S5. Comparison between (A) connectance C, (B) nestedness N, and (C) modularity Q values of snapshot networks (fine lines) and networks with links accumulated over longer periods (bold lines). Different line types (solid line, long-dashed line, and short-dashed line) correspond to different immigration rate values (respectively 10^{-4}; 10^{-3}; 10^{-2}). 30 replicates have been implemented for each immigration rate m value.
